# Draft Genome Sequence of *Pseudomonas fluorescens* Strain ET76, Isolated from Rice Rhizosphere in Northwestern Morocco

**DOI:** 10.1128/genomeA.00356-16

**Published:** 2016-05-19

**Authors:** Saida Aarab, Abdelhay Arakrak, Francisco Javier Ollero, Manuel Megías, Douglas Fabiano Gomes, Renan Augusto Ribeiro, Mariangela Hungria

**Affiliations:** a Equipe de Recherche de Biotechnologies et Génie des Biomolécules, Faculté des Sciences et Techniques de Tanger, Tanger, Morocco; b Departamento de Microbiología, Facultad de Biología, Universidad de Sevilla, Sevilla, Spain; c Embrapa Soja, Soil Biotechnology, Londrina, Paraná, Brazil; d Conselho Nacional de Desenvolvimento Científico e Tecnológico, Brasília, Distrito Federal, Brazil

## Abstract

*Pseudomonas fluorescens* ET76 was isolated from rice rhizosphere in northwestern Morocco. Its draft genome was estimated to be 6,681,652 bp with 5,789 coding sequences (CDSs). Genes encoding for type I to VI secretion systems, PvdQ, proteases, siderophores, hydrogen cyanide synthase, ACC-deaminase, among others, highlight its potential use in biological control of plant pathogens.

## GENOME ANNOUNCEMENT

*Pseudomonas fluorescens* strain ET76 was isolated from rhizosphere of rice (*Oryza sativa* L.) in paddies in northwestern Morocco. ET76 has typical properties of plant growth-promoting bacteria (PGPB), including the synthesis of ACC-deaminase and siderophores and the capacity of solubilizing phosphate (about 600 mg L^−1^), in addition to a strong proteolytic activity *in vitro*. The biotechnological properties of ET76 are highlighted by its antagonist activity to important plant pathogens, including both bacteria (*Pseudomonas savastanoi, Clavibacter michiganensis*, *Xanthomonas campestris* var. *citri*, and *Ralstonia solanacearum*) and fungi (*Fusarium oxysporum*, *Phytophthora cactorum*, *Phytophthora cinnamomi*, *Botryotinia fuckeliana*, *Verticillium dahliae*, and *Colletotrichum acutatum*); therefore, the strain has the potential to be used as an inoculant (1).

To access the bacterial genome sequence of ET76, total DNA was extracted using the DNeasy blood and tissue kit (Qiagen) and processed on the MiSeq sequencing system (Illumina) at Embrapa Soja, Londrina, Brazil. Shotgun sequencing generated a genome coverage of about 19-fold. Sequences were submitted to the Rapid Annotation using Subsystem Technology (RAST) server (2) and the genome was estimated to be 6,681,652 bp, assembled in 68 contigs. Annotation in RAST identified 5,789 coding sequences (CDSs), of which 52% were classified in 545 subsystems. Most of the categories with CDSs classified into subsystems were amino acids and derivatives (15.7%); carbohydrates (11.2%); and cofactors, vitamins, prosthetic groups, and pigments (8.2%). The highest genome similarity was found with *Pseudomonas fluorescens* strain Q8r1-96.

Genes encoding for type I, II, III, IV, V, VI, and VIII secretion systems and for the acylase PvdQ involved in quorum quenching were detected, but not for AHL synthase. Noteworthy was the high number of proteases, such as subtilin-like serine protease, collagenase and related proteases, and metallo proteases and cysteine proteases; in addition, there were several lipases and phospholipases. In relation to the chemotaxis and motility category, there are 202 genes (4.2% of those classified in subsystems), including genes for switching motility such as *pilHGT*. ET76 also carries a large number of genes (221, 5.0%) related to stress response, 95 of which are involved in oxidative stress.

It has been reported that the antimicrobial activity of *Pseudomonas* spp. may be related to the synthesis of metabolites such as 2, 4-diacetylphloroglucinol (2, 4-DAPG), pyrrolnitrin, pyoluteorin, or hydrogen cyanide (3, 4). The genome of ET76 carries genes related to the biosynthesis of pyrroloquinoline quinone, phenazine, the siderophore pyoverdine, and for hydrogen cyanide synthase (*hcnABC* genes). The presence of these molecules could explain the antagonist activity of ET76 to pathogenic bacteria and fungi, and reveals a great potential for use as a biological control agent. In addition, we confirmed that the genome carries genes coding for the enzyme ACC(1-aminocyclopropane-1-carboxylate)-deaminase, which metabolizes ACC, a precursor of the ethylene biosynthesis in higher plants, which could help plant growth and development under stress conditions by reducing the stress related to ethylene (5, 6).

### Nucleotide sequence accession numbers.

This whole-genome shotgun project has been deposited at DDBJ/EMBL/GenBank under the accession number LNAB00000000, SUBID SUB1165101, BioProject PRJNA301338, BioSample SAMN04244127, version 02.

## References

[1] Hungria M, Loureiro MF, Mendes IC, Campo RJ, Graham PH 2005 Inoculant preparation, production and application, p 223–254. *In* Werner W, Newton WE (ed), Nitrogen fixation in agriculture, forestry, ecology and the environment. Springer Verlag, Dordrecht. doi:10.1007/1-4020-3544-6_11.

[2] Aziz RK, Bartels D, Best AA, DeJongh M, Disz T, Edwards RA, Formsma K, Gerdes S, Glass EM, Kubal M, Meyer F, Olsen GJ, Olson R, Osterman AL, Overbeek RA, McNeil LK, Paarmann D, Paczian T, Parrello B, Pusch GD, Reich C, Stevens R, Vassieva O, Vonstein V, Wilke A, Zagnitko O 2008 The RAST server: Rapid Annotations using Subsystems Technology. BMC Genomics 9:75. doi:10.1186/1471-2164-9-75. 18261238 PMC2265698

[3] Fischer S, Prıncipe A, Alvarez F, Cordero P, Castro M, Godino A, Jofré E, Mori G 2013 Fighting plant diseases through the application of *Bacillus* and *Pseudomonas* strains, p 165–193. *In* Aroca R (ed), Symbiotic endophytes: Soil biology, vol 37 Springer Verlag, Berlin. doi:10.1007/2F978-3-642-39317-4_9.

[4] Paulsen IT, Press CM, Ravel J, Kobayashi DY, Myers GS, Mavrodi DV, DeBoy RT, Seshadri R, Ren Q, Madupu R, Dodson RJ, Durkin AS, Brinkac LM, Daugherty SC, Sullivan SA, Rosovitz MJ, Gwinn ML, Zhou L, Schneider DJ, Cartinhour SW, Nelson WC, Weidman J, Watkins K, Tran K, Khouri H, Pierson EA, Pierson LS, Thomashow LS, Loper JE 2005 Complete genome sequence of the plant commensal *Pseudomonas fluorescens* Pf-5. Nat Biotechnol 23:873–878. doi:10.1038/nbt1110. 15980861 PMC7416659

[5] Glick BR 2014 Bacteria with ACC deaminase can promote plant growth and help to feed the world. Microbiol Res 169:30–39. doi:10.1016/j.micres.2013.09.009. 24095256

[6] Saleem M, Arshad M, Hussain S, Bhatti AS 2007 Perspective of plant growth promoting rhizobacteria (PGPR) containing ACC deaminase in stress agriculture. J Ind Microbiol Biotechnol 34:635–648. doi:10.1007/s10295-007-0240-6. 17665234

